# Presence of increased inflammatory infiltrates accompanied by activated dendritic cells in the left atrium in rheumatic heart disease

**DOI:** 10.1371/journal.pone.0203756

**Published:** 2018-09-27

**Authors:** Mikio Shiba, Yasuo Sugano, Yoshihiko Ikeda, Hideshi Okada, Toshiyuki Nagai, Hatsue Ishibashi-Ueda, Satoshi Yasuda, Hisao Ogawa, Toshihisa Anzai

**Affiliations:** 1 Department of Cardiovascular Medicine, National Cerebral and Cardiovascular Center, Osaka, Japan; 2 Department of Cardiovascular Medicine, Osaka University Graduate School of Medicine, Osaka, Japan; 3 Departement of Cardiovascular Medicine, Keiyu Hospital, Yokohama, Japan; 4 Department of Pathology, National Cerebral and Cardiovascular Center, Osaka, Japan; 5 Department of Emergency and Disaster Medicine, Gifu University Graduate School of Medicine, Gifu, Japan; 6 Department of Cardiovascular Medicine, Hokkaido University Graduate School of Medicine, Sapporo, Japan; University of Miami School of Medicine, UNITED STATES

## Abstract

**Aims:**

Left atrial (LA) structural remodelling develops in rheumatic heart disease (RHD) according to the disease severity of the mitral valve and the presence of atrial fibrillation. Sustained active inflammation has been previously reported in the LA of patients with RHD, suggesting a direct role of cell-mediated immunity in the pathogenesis of LA remodelling. Dendritic cells (DCs) have a major antigen-presenting role, and are known as crucial modulators of innate and adaptive immunity. We investigated whether DCs are involved in the pathogenesis of LA remodelling in RHD.

**Methods and results:**

Immunohistochemical analyses were performed using antibodies to CD11c, CD209 and CD80 as markers of myeloid DCs, migratory-active DCs, mature DCs and infiltrated inflammatory cells including T lymphocytes (CD3) and M1 (CD68; pro-inflammatory profile) and M2 (CD163; pro-resolution profile) macrophages. Furthermore, tenascin-C, an extracellular matrix (ECM) protein that appears during ECM remodelling and inflammatory response, was examined. Infiltrated myeloid DCs, migratory-active DCs, mature DCs and other inflammatory infiltrates including T lymphocytes and M1 and M2 macrophages, were significantly higher in the RHD group than the non-RHD group. The positive area fraction for tenascin-C was significantly higher in the RHD group than in the non-RHD group.

**Conclusion:**

Our histological findings suggest that inflammation may persist long after a bout of rheumatic fever, ultimately leading to ECM remodelling. We identified and quantitatively assessed several subsets of DCs and other immunocompetent cells, and our results indicated that activation of DCs has some role in persistence of LA inflammation in patients with chronic RHD.

## Introduction

Rheumatic fever (RF) and the resultant rheumatic heart disease (RHD) are caused by a preceding acute group A streptococcal infection, and remain highly prevalent, particularly in the developing world.[1,2] The chronic inflammatory reaction associated with RHD possibly induces tissue fibrosis, resulting in the development of valve stenosis and/or insufficiency that may require valve surgery.[3] Serious adverse clinical events such as heart failure, stroke and death can result during the course of RHD; therefore, RHD is associated with major medical and social problems worldwide.[4] The repeated or ongoing autoimmune inflammatory response against self-antigens is considered to be involved in the pathogenesis of RHD.[5]

Besides valve impairment of RHD, increased risk of atrial fibrillation (AF) has been reported, possibly in association with repeated or ongoing inflammation in the left atrium (LA).[6,7] The presence of persistent inflammatory activity in the atrial myocardium in RHD may be an additional risk factor for the development of AF.[8,9] Enlargement of the LA, which is known as ‘giant LA’ on the rare occasion when it reaches an extraordinary size, has been found in some patients with RHD.[10] In addition to the excessive haemodynamic burden on the LA, long-standing autoimmune-based inflammation as a result of RHD could be a possible factor contributing to LA remodelling. Activation of T lymphocytes in the presence of antigen-presenting cells such as macrophages and dendritic cells (DCs) has been reported to be involved in the pathogenesis of inflammation in RHD.[11,12] However, no evidence has been presented showing that LA tissue remodelling develops according to such modulators of innate and adaptive immunity and the subsequent extracellular matrix (ECM) remodelling.

To clarify whether activation of DCs would be involved in the LA remodelling process in RHD, we used immunostaining with specific antibodies to immune-associated cells to indicate the direct infiltration of these cells using LA specimens harvested from human autopsies on AF patients with and without RHD. Furthermore, fibrosis and the accumulation of tenascin-C, an immune-related ECM protein,[13] were semi-quantitatively measured to assess ECM remodelling.

## Materials and methods

### Patients

Written informed consent was obtained for all subjects. Formalin-fixed autopsy specimens from the *National Cerebral and Cardiovascular Center Autopsy Archives* were taken from consecutive RHD subjects from 2002 to 2014 (RHD group, n = 5) and from age-, sex- and LA diameter–matched AF subjects without RHD (non-RHD group, n = 5). The diagnosis of RHD was made according to both a past history of RF and coexisting valvular disease morphologically consistent with rheumatic change. All patients in the RHD and non-RHD groups exhibited chronic persistent AF. Tissue samples were taken from the posterior wall in the LA area of the autopsied hearts. For conventional histochemistry, paraffin-embedded tissues were cut into 4-μm-thick slices and observed with hematoxylin and eosin (H&E) staining and Masson’s trichrome staining. Information about the patients’ baseline characteristics, such as age, gender, body mass index, heart rate, heart rhythm, past history and laboratory data was collected from their medical records. The patients’ characteristics and laboratory data were obtained retrospectively within 6 months from their last admission. Laboratory data were obtainable in a stable condition in all patients. Echocardiography was performed during the last admission in all patients. The parameters were measured according to the recommendations of the American Society of Echocardiography.[14] The left ventricular end-diastolic diameter, left ventricular end-systolic diameter and left atrial (LA) diameter were obtained from the M-mode or parasternal long axis views. The percentage of ejection fraction was calculated using the Teichholz formula.[15]

Autopsies were performed as soon as possible, within 9 h for all patients, after death, and informed consent was obtained from family members of all the subjects. This study was approved by the Research Ethics Review Committee of National Cerebral and Cardiovascular Center, and conforms to the principles of the Declaration of Helsinki.

### Immunohistochemical staining

Immunohistochemistry (IHC) was performed following established methods using buffered formalin-fixed and paraffin-embedded (FFPE) sections and antibodies to infiltrated inflammatory cells, including T lymphocytes (CD3), M1 (CD68; pro-inflammatory profile) and M2 (CD163; pro-resolution profile) macrophages, myeloid DCs (CD11c), migratory-active DCs (CD209) and mature DCs (CD80). Additionally, tenascin-C, one of the ECM proteins activated in ECM remodelling and the inflammatory response, was examined. IHC was performed on 4-μm-thick FFPE sections from the LA wall. We used a fully automated IHC and *in situ* hybridisation staining system, Bond-III (Leica Biosystems, Wetzlar, Germany). In brief, specimens were deparaffinised, and the antigen was retrieved on the instrument. All slides were incubated with primary antibodies detecting CD3 (#N1580, diluted 1:10; Dako, Glostrup, Denmark), CD68 (#M0814, diluted 1:1000; Dako), CD163 (#ab74604, diluted 1:10; Abcam, Cambridge, UK), CD11c (#EP1347Y, diluted 1:100; GeneTex, Irvine, CA, USA), CD209 (Clone DCN46, #551249, diluted 1:1000; BD Biosciences, Franklin Lakes, NJ, USA), CD80 (#MAB140, diluted 1:60; R&D Systems, Minneapolis, MN, USA) and tenascin-C (4F10TT clone, #10337, diluted 1:1000, with 24-h decalcification with formic acid and citric acid; Immuno-Biological Laboratories, Gunma, Japan). Antibody binding was visualised using the avidin–biotin complex method according to the manufacturer’s instructions (Vectastain ABC; Vector Laboratories, Burlingame, CA, USA). The primary antibody was omitted from these protocols as a negative control. The sections were subsequently counterstained with haematoxylin.

### Analyses of the inflammatory cell infiltrates and tenascin-C expression

IHC-positive infiltrating cells were counted in the area of each sample at a magnification of 100× in 10 randomly selected fields as previously described by two pathologists (Y.I. and H.I-U.) in a blinded manner.[16] Positive cells in the vessels were excluded from the total count. Finally, the number of positive cells /0.1 mm^2^ was calculated. The area fraction of tenascin-C was semi-quantitatively measured as the percentage of the tenascin-C-positive area using Image J software (National Institutes of Health, Bethesda, MD, USA) as previously described.[17]

### Statistical analysis

Data were presented as the mean ± SD for continuous variables or as numbers (percentage) for categorical variables. Continuous variables were compared using Fisher’s exact test or the Wilcoxon rank-sum test. Pearson’s correlation analysis was performed to estimate correlation coefficients. A p value of less than 0.05 was considered statistically significant, and all statistical analyses were performed using SPSS Statistics version 23.0 (IBM Corp., Armonk, NY, USA).

## Results

### Patients’ characteristics

The background characteristics of all patients in this study are shown in Table 1. All patients in the RHD group underwent mitral valve replacement for mitral valve disease. All patients in both the RHD and non-RHD groups were on warfarin. None of the patients were diagnosed with myocarditis using histopathologic Dallas criteria and none had a significant neutrophil infiltration. Table 2 shows the demographic, laboratory and echocardiographic data for the patients. The age, sex and LA dimensions of the non-RHD group were matched with those of the RHD group. Dyslipidaemia was less common in the RHD group (p = 0.04); otherwise, there were no significant differences between the groups, including cardiac dimension and contractile function as assessed by echocardiography.

**Table 1:** Individual baseline characteristics.

Patient	Age, yrs	Sex	Valvular disease	Operation	Rhythm	Rate, bpm	AF duration, yrs	Cause of death
RHD group								
1	68	M	MS	MVR	AF	72	52	ADHF + Pneumonia
2	78	M	MS, ASR	DVR	AF	54	26	Cerebral haemorrhage
3	64	M	MS, AS	DVR	AF	92	28	ADHF
4	70	F	MS	MVR	AF	85	26	ARDS + Sepsis
5	75	M	MSR, AR	DVR	AF	75	40	ADHF
Non-RHD group								
6	71	M	N/A	N/A	AF	44	21	Pneumonia
7	67	M	N/A	N/A	AF	62	17	Cerebral infarction
8	75	M	N/A	N/A	AF	60	27	AAA rupture
9	84	F	N/A	N/A	AF	98	15	Cerebral infarction
10	77	F	AR	AVR	AF	107	40	Cerebral infarction

RHD indicates rheumatic heart disease; M, male; F, female; MS, mitral stenosis; ASR, aortic stenosis and regurgitation; AS, aortic stenosis; MSR, mitral stenosis and regurgitation; AR, aortic regurgitation; MVR, mitral valve replacement; DVR, double valve replacement; AVR, aortic valve replacement; AF, atrial fibrillation; ADHF, acute decompensated heart failure; ARDS, acute respiratory distress syndrome; AAA, abdominal aortic aneurysm; N/A, not applicable.

**Table 2:** Demographic, laboratory and echocardiographic data.

	Overall (n = 10)	RHD group (n = 5)	Non-RHD group (n = 5)	*P* value
**Demographic data**				
**Age, years**	73 ± 6	71 ± 6	75 ± 6	0.40
**Male**	7 (70)	4 (80)	3 (60)	0.49
**Body mass index, kg/m^2^**	19.5 ± 4.4	18.9 ± 2.5	20.2 ± 6.0	0.35
**Heart rate, bpm**	75 ± 20	76 ± 14	74 ± 27	0.92
**AF rhythm**	10 (100)	5 (100)	5 (100)	
**Duration of AF, years**	29 ± 12	34 ± 11	24 ± 10	0.14
**Systolic BP, mmHg**	131 ± 28	116 ± 21	145 ± 28	0.17
**Diastolic BP, mmHg**	77 ± 23	67 ± 16	86 ± 27	0.29
**Hypertension**	8 (80)	3 (60)	5 (100)	0.11
**Dyslipidaemia**	3 (30)	0 (0)	3 (60)	0.04
**Diabetes mellitus**	1 (10)	0 (0)	1 (20)	0.29
**Current smoker**	2 (20)	0 (0)	2 (40)	0.11
**Laboratory data**				
**WBC, ×10^3^/μL**	6.4 ± 1.2	5.9 ± 1.1	6.9 ± 1.1	0.17
**RBC, ×10^4^/μL**	351 ± 67	320 ± 61	383 ± 64	0.08
**HGB, g/dL**	11.6 ± 2.5	10.7 ± 2.0	12.4 ± 2.8	0.25
**AST, IU/L**	41 ± 21	42 ± 28	41 ± 13	0.46
**ALT, IU/L**	21 ± 8	23 ± 9	19 ± 6	0.35
**BUN, mg/dL**	44 ± 17	43 ± 13	45 ± 21	0.92
**Creatinine, mg/dL**	1.80 ± 1.30	1.83 ± 1.61	1.76 ± 1.12	0.92
**CRP, mg/dL**	0.83 ± 0.90	0.56 ± 0.77	1.09 ± 1.04	0.25
**Echocardiographic data**				
**LVEDD (mm)**	52 ± 15	60 ± 18	44 ± 7	0.12
**LVESD (mm)**	39 ± 18	48 ± 23	30 ± 8	0.21
**EF (%)**	47 ± 19	41 ± 25	52 ± 9	0.60
**LAD (mm)**	57 ± 10	57 ± 9	56 ± 11	0.68

RHD indicates rheumatic heart disease; AF, atrial fibrillation; BP, blood pressure; WBC, white blood cells; RBC, red blood cells; HGB, haemoglobin; AST, aspartate aminotransferase; ALT, alanine aminotransferase; BUN, blood urea nitrogen; CRP, C-reactive protein; LVEDD, left ventricular end-diastolic diameter; LVESD, left ventricular end-systolic diameter; EF, ejection fraction; LAD, left atrial diameter.

Values are the mean ± SD or n (%).

### Representative pathological findings

**Fig 1:**
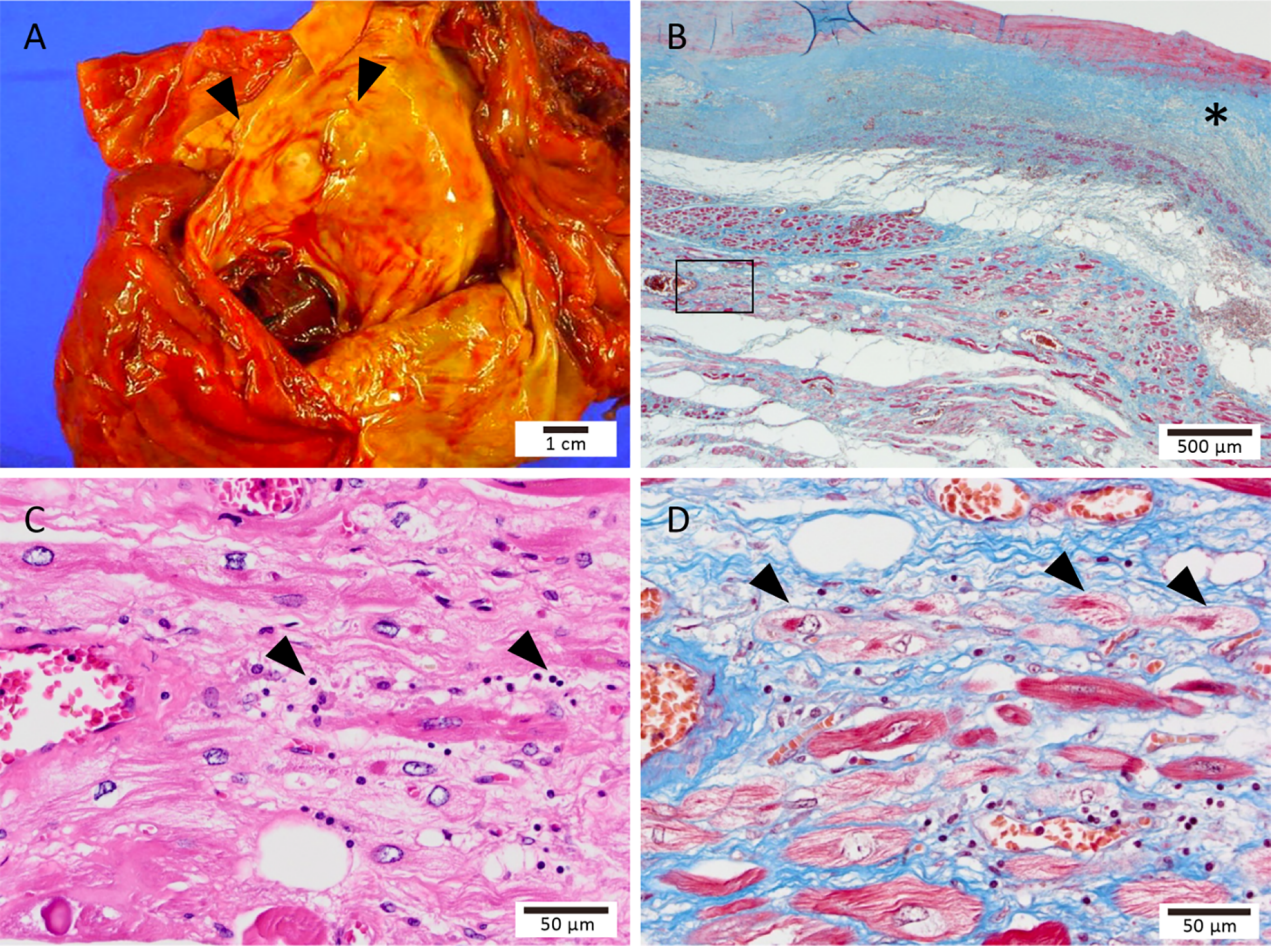
Macroscopic and microscopic autopsy findings in a patient with rheumatic heart disease. (A) Photomacrograph showing a notably thickened left atrial endocardium with fibrotic lesions (arrowheads). (B) Photomicrograph of a left atrial myocardium section stained with Masson’s trichrome. The left atrial wall shows diffuse interstitial and replacement fibrosis with endocardial thickening (*). (C) High-power field image of the square frame in (B) stained with hematoxylin and eosin. Lymphocytes infiltrating predominantly into the myocardial interstitium with replacement fibrosis (arrowheads). (D) Same as the (C) area stained with Masson’s trichrome showing myofibrillar degeneration (arrowheads).

The representative macroscopic and micrographic LA findings on autopsy in the RHD group are depicted in Fig 1. The dilated LA endocardial wall was diffusely thickened with fibrocalcific lesions (Fig 1A). The histopathology of the LA myocardium showed a multifocal interstitial inflammatory process with extensive replacement fibrosis and residual cardiomyocyte degeneration. In addition, dystrophic calcification appeared on the endocardial surfaces of the LA. Remarkably, lymphocyte infiltration was observed predominantly in the myocardial interstitium with replacement fibrosis (Fig 1B and 1C). Myofibrillar degeneration, a form of ongoing myocardial damage, was present in the area of lymphocyte infiltration (Fig 1D). Further, no giant cells, vasculitis, or microorganisms were identified.

### Counts of inflammatory infiltrates

**Fig 2:**
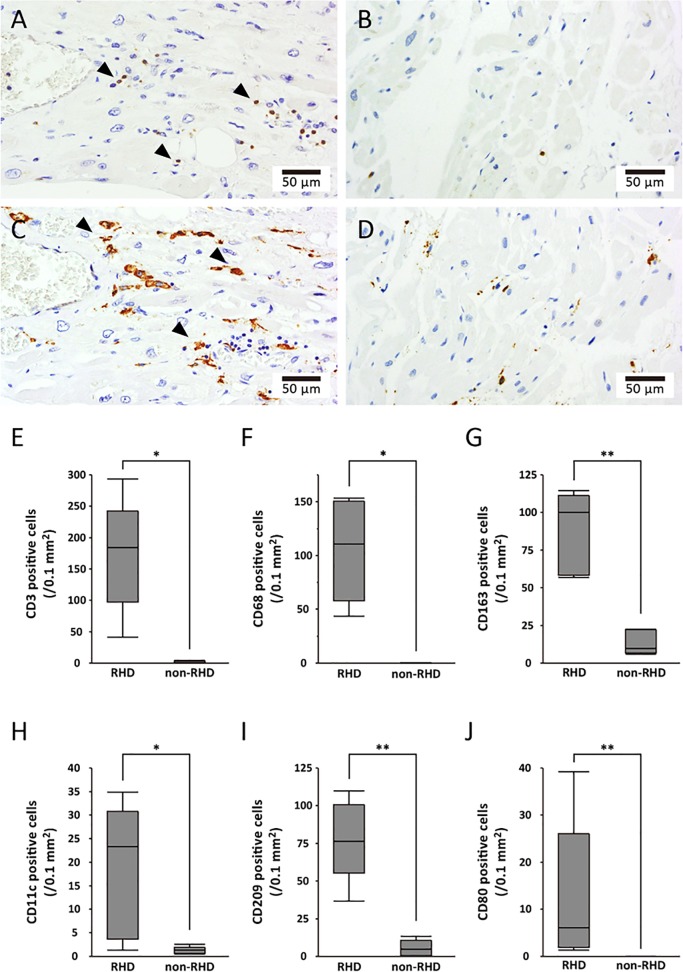
Immunohistochemical staining of left atrial myocardial section. Photomicrographs of a left atrial myocardium section stained immunohistochemically for CD3 (A, B) and CD209 (C, D). The myocardium shows intense mixed infiltrate composed of CD3-positive T lymphocytes (A, arrowheads) and CD209-positive migratory-active dendritic cells (C, arrowheads) without obvious necrosis of the cardiomyocytes in the RHD group in contrast to the non-RHD group (B, D). The graph shows the counts of immunohistochemical staining for the RHD and non-RHD groups. The numbers of infiltrated CD3- (E), CD68- (F), CD163- (G), CD11c- (H), CD209- (I) and CD80- (J) positive cells are also shown. * p < 0.05, ** p < 0.01.

Representative photomicrographs of the IHC for patients in the RHD group (Fig 2A, CD3-positive T lymphocytes; Fig 2C, CD209-positive migratory-active DCs) and non-RHD group (Fig 2B, T lymphocytes; Fig 2D, migratory-active DCs) are shown. High counts of both T lymphocytes and CD209-positive DCs were detected in the interstitium in the RHD group, whereas there were few IHC- positive cells in the non-RHD group. Similar results were observed for the other immunostaining tests, including CD68 (M1 macrophage), CD163 (M2 macrophage), CD11c (myeloid DC) and CD80 (mature DC). Statistical analyses revealed that the numbers of infiltrated T lymphocytes (173 ± 91 vs. 2 ± 1 /mm^2^, p = 0.012, Fig 2E), M1 macrophages (106 ± 47 vs. 0.1 ± 0.2 /mm^2^, p = 0.010, Fig 2F), M2 macrophages (88 ± 27 vs. 14 ± 8 /mm^2^, p = 0.008, Fig 2G), myeloid DCs (18 ± 14 vs. 1 ± 1 /mm^2^, p = 0.035, Fig 2H), migratory-active DCs (78 ± 27 vs. 5 ± 5 /mm^2^, p = 0.008, Fig 2I) and mature DCs (12 ± 16 vs. 0 ± 0 /mm^2^, p = 0.008, Fig 2J) were significantly higher in the RHD group than in the non-RHD group.

### Tenascin-C-positive area

**Fig 3:**
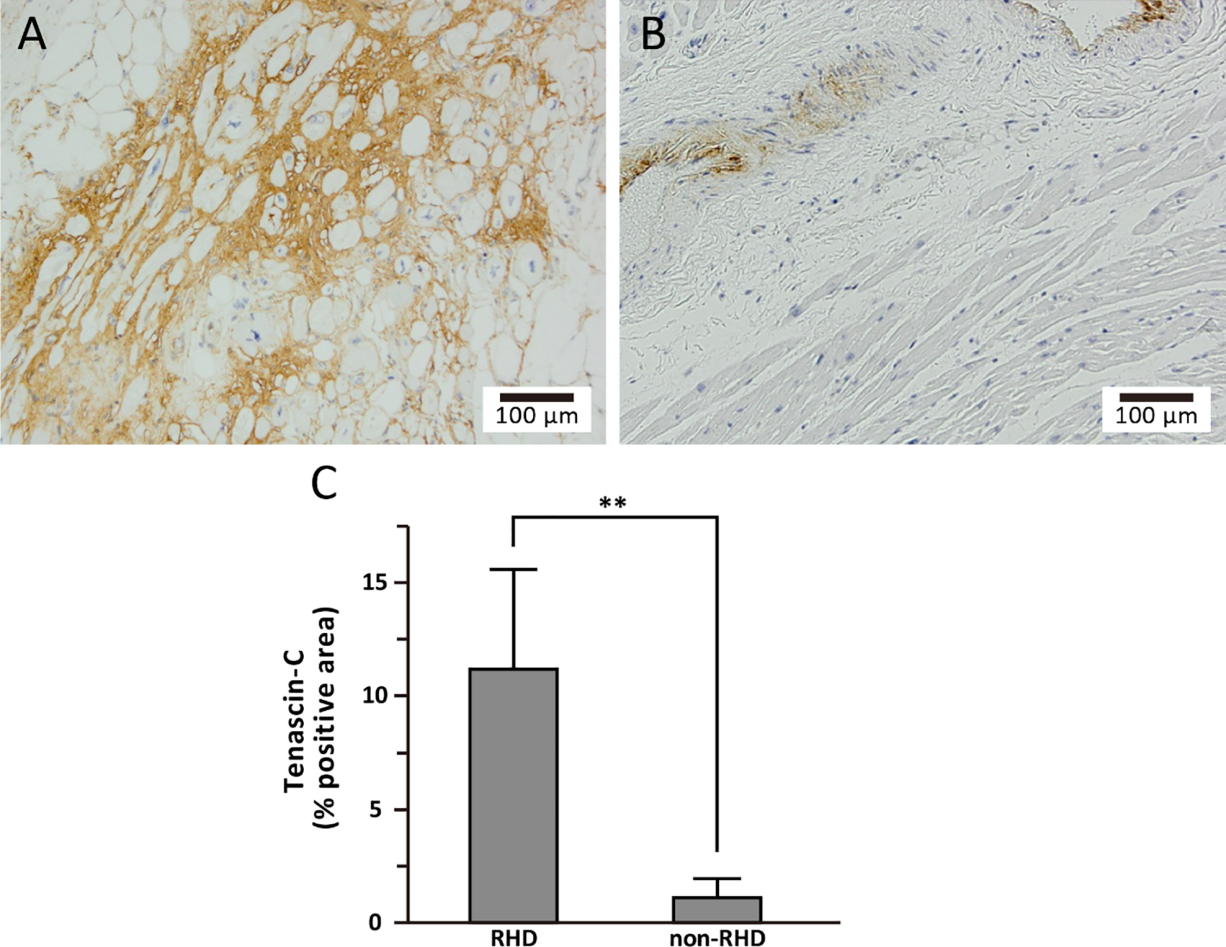
Expression of tenascin-C in left atrial myocardium section. Photomicrographs of a LA myocardium section stained immunohistochemically for tenascin-C in an RHD patient (A) and a non-RHD patient (B). The graph shows the percentage of the tenascin-C-positive area in the two groups (C). Expression of tenascin-C was significantly greater in the RHD group compared to the non-RHD group. ** p < 0.01.

**Fig 4:**
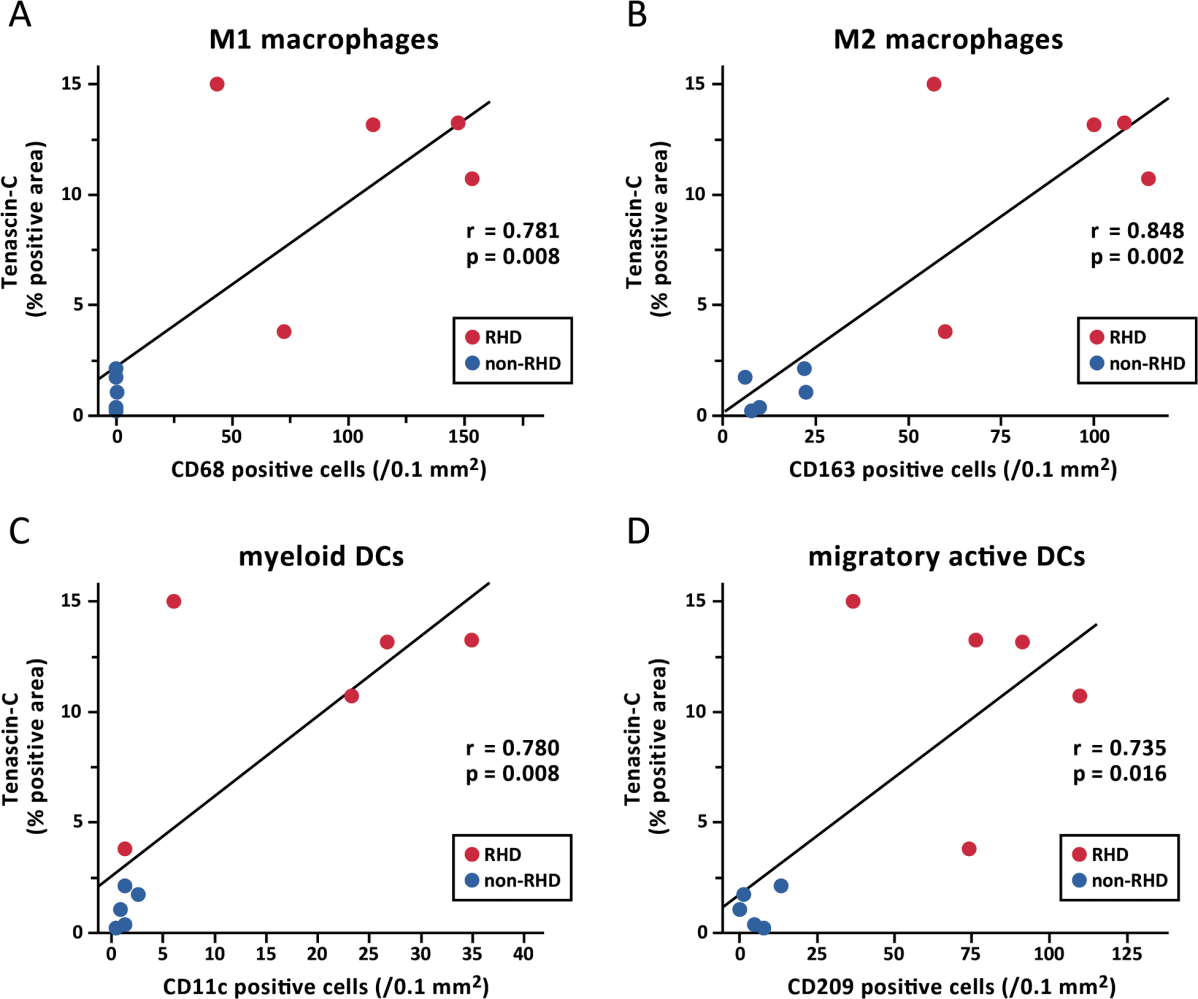
Correlation between tenascin-C positive area and myocardial infiltrates. There was significant correlation between the tenascin-C-positive area and each of the numbers of CD68-positive M1 macrophages (A, r = 0.781, p = 0.008), CD163-positive M2 macrophages (B, r = 0.848, p = 0.002), CD11c-positive myeloid DCs (C, r = 0.780, p = 0.008) and CD209-positive migratory-active DCs (D, r = 0.735, p = 0.016).

Representative positive IHC images of the tenascin-C-on LA sections for the RHD and non-RHD groups are shown in Fig 3A and 3B, respectively. The tenascin-C-positive area was significantly larger in the RHD group than in the non-RHD group (11.2 ± 4.4 vs. 1.1 ± 0.8%, p = 0.008, Fig 3C). The tenascin-C-positive area was significantly correlated with the numbers of M1 macrophages (CD68, r = 0.781, p = 0.008, Fig 4A), M2 macrophages (CD163, r = 0.848, p = 0.002, Fig 4B), myeloid DCs (CD11c, r = 0.780, p = 0.008, Fig 4C) and migratory-active DCs (CD209, r = 0.735, p = 0.016, Fig 4D).

## Discussion

The present study demonstrated more infiltrating inflammatory cells, including T lymphocytes, macrophages and DCs, in the LA in RHD patients than in that of age-, sex- and LA size–matched non-RHD patients, which suggests that antigen presentation and the consequent inflammatory activation remains in the enlarged LA in RHD. Furthermore, the increased expression of tenascin-C suggests that LA remodelling may develop according to ECM remodelling with tenascin-C in response to the inflammatory cell activation. Our findings accentuate the key pathophysiological aspect of RHD that persistent immune responses play a major role in the development of LA structural remodelling in RHD.

We initially focused on the DCs that are known to be key antigen-presenting cells that orchestrate innate and adaptive immunity in the relevant tissues.[18] DCs function by both initiating the inflammatory cascade and regulating the subsequent inflammatory response.[19] Because post-streptococcal autoimmune activation is considered to be involved in the pathophysiological development of RHD, we conducted this study to identify the migration and activation of DCs in the LA of RHD patients. Our results revealed a significant increase in several phenotypes of DCs, along with increases in substantial inflammatory cells such as macrophages and T lymphocytes, in the LA of patients with RHD. Based on this finding, we suggest that an immune response may be triggered as a result of antigen presentation by DCs and activate macrophages and T lymphocytes in the course of LA remodelling in RHD.

In the present study, all patients with RHD exhibited an enlarged LA and AF. Structural LA remodelling in AF is complex, with several mechanisms, including inflammation, oxidative stress, mechanical stretching and ischaemia.[20] LA haemodynamic stretch itself could induce immune activation and contribute to ECM remodelling. Previously, histological analysis of atrial biopsies in patients with AF showed that two-thirds of the patients presented inflammatory infiltration adjacent to necrotic myocytes, suggesting that inflammation may be involved in atrial remodelling in AF patients.[21] In this study, however, we compared LA tissues obtained from RHD patients with those obtained from LA size–matched non-RHD patients, and our results showed markedly higher counts of immunocompetent cells, including several phenotypic subsets of DCs, in RHD patients compared with non-RHD patients. This finding suggests that RHD-derived remodelling rather than haemodynamic-related stress would be associated with LA remodelling in RHD.

We also investigated tenascin-C, which plays an important role in ECM remodelling. Immunohistochemical study demonstrated that tenascin-C is strongly expressed in the LA interstitium in RHD. Tenascin-C is a large extracellular glycoprotein that is only expressed during embryonic development, and not in the normal adult heart.[22] However, with exposure to inflammatory stress, it reappears in the heart in, for instance, myocardial infarction and myocarditis.[23] Recently, the close association between tenascin-C and inflammation has been uncovered. [24,25] Patients with RHD exhibit increased expression of tenascin-C as well as increased inflammation; as stated above, tenascin-C is strongly associated with tissue remodelling. Thus, we believe that tenascin-C expression may be key to LA remodelling due to inflammation. In this study, we demonstrated that tenascin-C was strongly correlated with inflammatory infiltrates including M1 and M2 macrophages, myeloid DCs and migratory-active DCs. This finding suggests that activation of macrophages and DCs might be involved in ECM remodelling in RHD. We speculate that autoimmune-associated mechanisms would raise the DCs activation first, followed by macrophage activation to both M1 and M2, which would develop ECM remodelling associated with tenascin-C synthesis. Interestingly, the strongest correlation was with CD163 for M2 macrophages (pro-resolution profile), which correspond to our recent finding that M2 macrophages are crucially associated with cardiac remodelling in dilated cardiomyopathy.[26] Further, increased numbers of DCs and other inflammatory cells in LA were associated with ECM remodelling, which may affect the persistent inflammatory process in RHD.

### Limitations

There are several limitations in this study. First, this was a single-centre study with a retrospective design. A limited chance to conduct autopsy of RHD patients provided small sample size for this study, although all subjects with RHD from 2002 to 2014 were enrolled. Nevertheless, we obtained definitive data demonstrating prominent myocardial infiltration by DCs and other associated immune cells in LA of RHD patients and, thus, considered it worth reporting. Second, harvested tissue samples may degrade to some extent after myocardial blood supply stops, and, therefore, the reliability of immunohistochemistry may be decreased. To avoid this problem, we conducted autopsy as soon as possible, within 9 h of death for all subjects. Third, all patients in the RHD group had a history of mitral valve replacement with thoracotomy, which might have affected the myocardial inflammatory response and tenascin-C expression. However, one patient in the non-RHD group who had undergone a thoracotomy showed almost no inflammatory infiltration nor tenascin-C expression. Finally, we performed immunohistochemical staining with inflammatory and antigen-presenting cells using specific markers; thus, we were not able to show the antigen-presenting capacity of these cells nor their direct impact on atrial remodelling.

## Conclusions

Significant inflammatory cell infiltration such as DCs and macrophages of LA was noted in autopsied hearts with chronic RHD. Our quantitative analyses suggest that this immune activation may persist long after a bout of rheumatic fever and ultimately lead to ECM remodelling in association with expression of tenascin-C.

## Supporting information

S1 Table All relevant data about each patient. (XLSX)

S1 File Supplemental File_RHD20180709.xlsx. (XLSX)
